# Enhancing the Therapeutic Efficacy of Cancer Treatment With Cannabinoids

**DOI:** 10.3389/fonc.2018.00114

**Published:** 2018-04-24

**Authors:** Sayeda Yasmin-Karim, Michele Moreau, Romy Mueller, Neeharika Sinha, Raymond Dabney, Allen Herman, Wilfred Ngwa

**Affiliations:** ^1^Radiation Oncology, Brigham and Women’s Hospital, Boston, MA, United States; ^2^Dana–Farber Cancer Institute, Boston, MA, United States; ^3^Harvard Medical School, Boston, MA, United States; ^4^University of Massachusetts Lowell, Lowell, MA, United States; ^5^University Medical Center Mannheim, Heidelberg University, Mannheim, Germany; ^6^Cannabis Science, Inc., Irvine, CA, United States

**Keywords:** radiotherapy, cannabinoids or endocannabinoids, biomaterials, cancer, survival

## Abstract

Over the years, many *in vitro* and *in vivo* studies have shown the antineoplastic effects of cannabinoids (CBDs), with reports advocating for investigations of combination therapy approaches that could better leverage these effects in clinical translation. This study explores the potential of combination approaches employing CBDs with radiotherapy (RT) or smart biomaterials toward enhancing therapeutic efficacy during treatment of pancreatic and lung cancers. In *in vitro* studies, clonogenic assay results showed greater effective tumor cell killing, when combining CBDs and RT. Meanwhile, *in vivo* study results revealed major increase in survival when employing smart biomaterials for sustained delivery of CBDs to tumor cells. The significance of these findings, considerations for further research, and viable roadmap to clinical translation are discussed.

## Introduction

Many *in vitro* and *in vivo* studies have reported on the antitumorigenic effects of plant-derived cannabinoids (CBDs) and their synthetic analogs, including effects in inducing apoptosis and inhibiting tumor cell growth and metastasis (1, 2). Despite these reports of demonstrating the potential of CBDs as anticancer agents, clinical translation has been hampered in part by the need to demonstrate significant therapeutic efficacy with minimal toxicities or side effects. So far, the only published clinical trial on the use of CBDs for cancer treatment has been on a small pilot study involving intratumoral (IT) administration of CBDs in patients with recurrent glioblastoma multiforme (1, 3). This study and subsequent reports (1, 2) highlighted the need for further research to enhance the therapeutic efficacy of CBDs, including *via* combinations with other therapies like chemotherapy or radiotherapy (RT), or the use of other routes of administration that can better leverage the effects seen in preclinical *in vitro* and *in vivo* studies.

The advantage of combining CBDs with other therapies is that this may allow simultaneous targeting of tumor progression at different levels, while minimizing toxicities for these therapies relative to toxicities from higher doses when used as monotherapies. For example, studies suggest that fractionated RT can be a double-edged sword (4), killing tumor cells while also inducing tumor cell metastasis or invasion from the primary tumors. The use of CBDs in combination with RT could benefit from findings that CBDs can inhibit metastasis or invasion (5–8). CBDs have also been reported to enhance tumor cell apoptosis *via* increased generation of reactive oxygen species, which could enhance the DNA-damaging effects of RT (9). This could allow for the reduction of RT doses or fractions to minimize RT toxicities. Such a combination would be particularly beneficial for patients needing salvage RT but who are close to their RT normal tissue limits (10).

Meanwhile, the route of administration has a significant bearing on pharmacokinetics, the bioavailability, time course and hence effectiveness of CBDs as anticancer agents. The solubility of CBDs in water is poor, with inherent limits to intravenous and other routes of CBD delivery. Absorption following oral administration route for cancer treatment could also be limited by potential for CBD degradation by the acid of the stomach (11). Inhalation route exposes valid concerns about the adverse pulmonary effects this may have and limited effectiveness if not well targeted (12). It has been proposed that the administration of a low dose of CBDs directly to the tumor would increase effectiveness and reduce adverse side effects (13). The published pilot clinical trial (3) employed such an IT route with repeated daily administrations of CBDs. However, many studies suggest that sustained drug delivery approaches, e.g., using smart biomaterials can be more effective than repeated injections (12, 14–17).

Pancreatic and lung cancers are amongst the deadliest forms of cancers (18, 19), where greater effective treatment approaches are needed. Here, we explore the potential for enhancing the effectiveness of pancreatic and lung tumor cell kill *via*: (1) combination of CBDs with RT and (2) use of smart biomaterials loaded with CBDs for sustained *in situ* delivery. These findings are discussed with considerations toward clinical translation.

## Materials and Methods

### Cell Culture and Materials

Human lung cancer cell line A549 (ATCC) was maintained in RPMI media (GIBCO) supplemented with 10% FBS, 2 mmol/L l-glutamine, 1% penicillin, and streptomycin solution. Lewis lung cancer C57BL/6 back ground mouse cell line LLC-1 (ATCC) and pancreatic adenocarcinoma C57BL/6 back ground mouse cell line PANC-02 (ATCC) were maintained in DMEM media (GIBCO) supplemented with 10% FBS, 2 mmol/L l-glutamine, 1% penicillin, and streptomycin solution following standard protocol. All cells were maintained at 37°C in a humidified incubator under a 5% CO_2_ atmosphere following standard protocol. All supplements were obtained from Sigma-Aldrich and tissue culture plastics were obtained from Corning Life Sciences. All experimental cells were at least 95% alive.

### Clonogenic Cell Survival Assay

**Figure 1:**
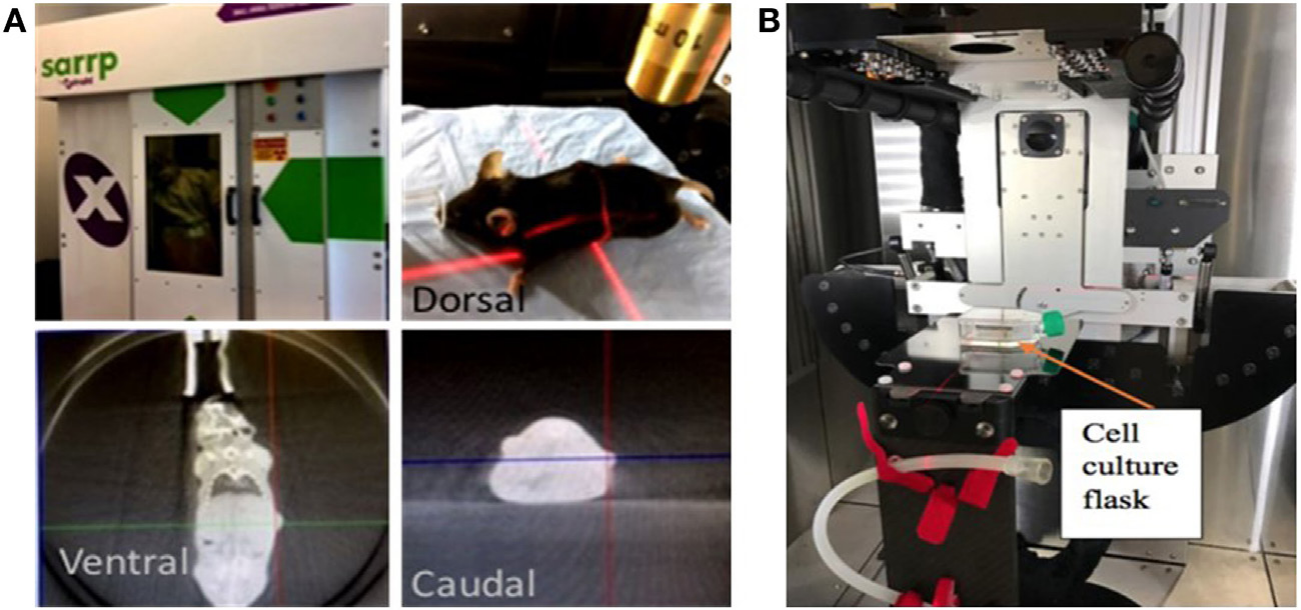
Small animal radiation research platform (SARRP). Showing pictures of set-up for **(A)** image-guided radiotherapy (RT) with computed tomography imaging done using 65 kVp photons at 0.5 mA, **(B)** *in vitro* studies with RT delivered using 220 kVp photons and 13 mA.

A549 cells from an actively growing monolayer were trypsinized and 300 cells per well were seeded in 12-well plates (Corning). After 24 h, seeded cells were treated with 0, 1, 2, and 5 μg/well of CBD concentrations. The cells were irradiated at 0, 2, and 4 Gy using 220 kVp energy, 13 mA, 24 h after the CBD treatment. A small animal radiation research platform (SARRP) (20, 21) was used for RT (Figure 1). The growing colonies (>50 cells/colony) were fixed with 75% ethanol and stained with 1% crystal violet (Sigma) 7–10 days after treatment. Colonies were counted using ImageJ software and a percent survival was calculated following standard protocol (22, 23).

### Smart Biomaterials

Smart radiotherapy biomaterials (SRBs) were prepared as described in previous work (14) using 200 mg of poly(lactic-co-glycolic) acid (Sigma-Aldrich) with MW 40,000–75,000 dissolved in 4 mL of Acetone. The resulting blend was loaded with a constant flow rate into a silicon tubing (VWR and Versilic) using a Harvard apparatus (Harvard Bioscience) and dried at 50°C for 72 h. The dried biomaterials were cut into 4 mm length to incorporate the CBD payload. CBD payloads were custom loaded into the SRBs with a unique potential for image-guided RT and sustained *in situ* delivery of the CBD payload. The SRBs are customizable allowing for loading of different concentration of CBD payloads tagged with fluorescence dye and the incorporation of high-Z nanoparticles (NPs) for enhanced computed tomography (CT) imaging contrast.

*In vitro* release of payloads was investigated by placing CBD-loaded SRBs into wells of a microplate, triplicated for each day the payload release was observed. The fluorescence intensity of the released payload was read using a microplate reader/photometer.

### Animal Studies

Immune uncompromised, wild-type (W+/+) C57BL/6 strain male and female mice were purchased from Jackson laboratory at the age of 8 weeks. All mice were maintained at Dana Farber Cancer Institute (DFCI) animal facility in accordance with institutional policies and Institutional Animal Care and Use Committee (IACUC)-approved guidelines. Mice between 10 and 12 weeks of age were used for the experiments. Syngeneic mouse models were created by subcutaneously (s.c.) implanting C57BL/6 mice with 2 × 10^5^ cells per tumor for PANC-02 cells for the pancreatic tumor model, and 5 × 10^4^ cells per tumor LLC-1 cells for the lung tumor model, in the flank of same background wild mice. For both cases, only the live cells were counted determined by Trypan blue staining.

Tumor growth was supervised regularly following DFCI animal protocol until a tumor size of approximately 6 mm in length was reached. Mice were then randomized into different cohorts. For the direct IT administration method, IT injection of CBD (0.1 mg, 5 mg/kg) dissolved in methanol was given using a 26 G insulin needle (BD Bioscience). Same volume of methanol was injected in control group tumors. For SRB-CBD treatment method, SRBs with the same dose of CBD as for IT method were administered to the tumors using clinical brachytherapy needles (IZI Medical Products). A control group of mice with tumors were also administered with SRBs loaded with same methanol concentration as CBD solution.

After treatment, a Vernier caliper was used to measure the length and width of the subcutaneous tumors. Tumor volumes were calculated as: (length × width^2^)/2. Dimension imaginary longitude to the leg was designated as length and the perpendicular was for width. The tumors were measured between the skin surface layers. The tumor volume was plotted against time.

Animal survival was performed for treatments following IACUC-approved protocol, which was predetermined based on published evidence justifying such a study design. Tumors reaching >1 cm in diameter or actively bleeding were determined as excessive tumor burden and mouse was euthanized following the protocol. For IT release studies, CT imaging was carried out using the SARRP (Figure 1) with 65 kVp and 0.5 mA. The CT imaging was conducted over 1 week.

### CT Image Analysis

The CT images were analyzed using the Preclinical Imalytics software. In the image analysis, the segmented tumor was made transparent to highlight the SRB. The results clearly depict the SRB disintegration in the tumor volume with lapse of time. The quantification of the images was performed using MATLAB software.

### Statistical Analysis

Log-rank (Mantel–Cox) test and Gehan–Breslow–Wilcoxon test were performed to analyze statistical significance of the survival assay for *in vivo* lung cancer model with GraphPad prism software.

## Results

**Figure 2:**
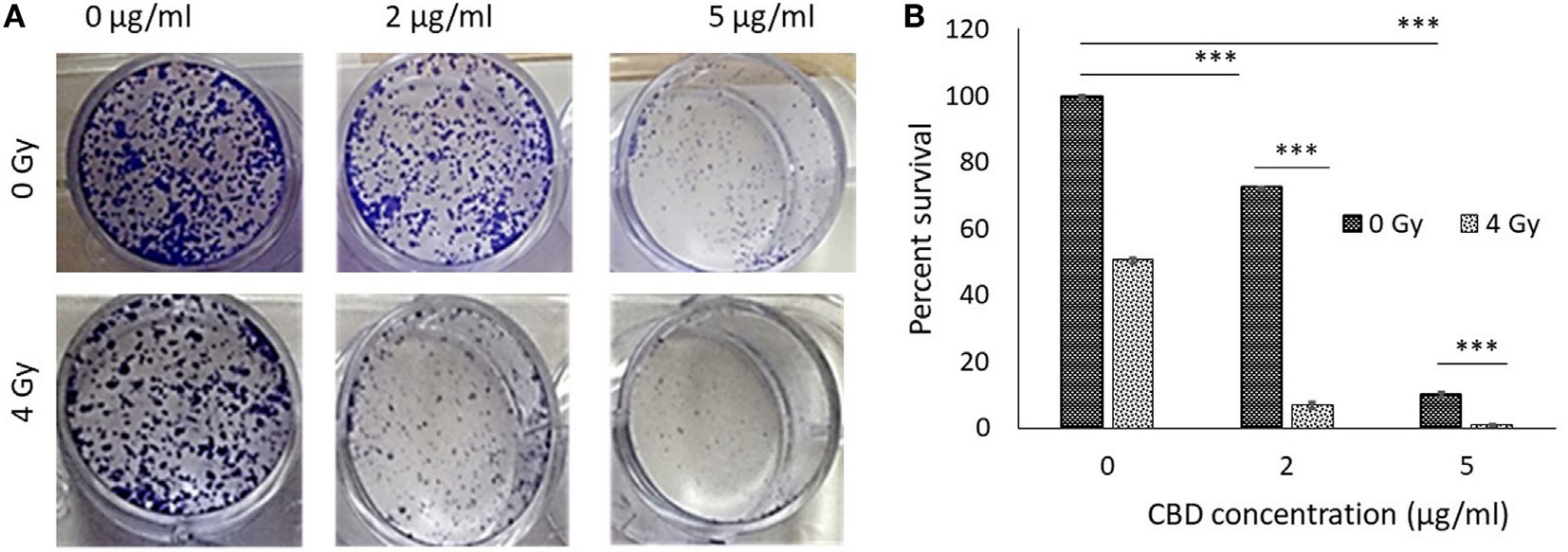
*In vitro* antitumor effect of cannabinoid (CBD). **(A)** Clonogenic assay results comparing results for different CBD radiotherapy (RT) dose combinations. **(B)** Perspective results of synergistic outcomes when combining RT at 4 Gy with different CBD doses.

Clonogenic assay results are shown in Figures 2A,B demonstrating substantially enhanced tumor cell killing when using CBDs with RT. Significant synergy is observed in the study arm combining 2 µg of CBD with RT at 4 Gy. Such synergy may allow for greater effective tumor cell killing while reducing the dose of RT. Remarkably, 5 µg of CBD was found to achieve greater tumor cell killing than 4 Gy of RT. This supports findings in previous studies that CBDs can induce apoptosis, with potential mechanism being the generation of highly potent reactive oxygen species (9). This effect combined with DNA damage by RT could account for the observed synergistic outcomes.

The remarkable *in vitro* study results with and without RT followed an exposure of the tumor cells to CBD over a prolonged period of 24 h. With view to translating such effective tumor cell killing *in vivo*, we hypothesized that using smart biomaterials for sustained/prolonged delivery of CBDs in tumors can also enhance the effectiveness of *in vivo* tumor cell kills with CBDs. To test this hypothesis, CBD was loaded into SRBs.

**Figure 3:**
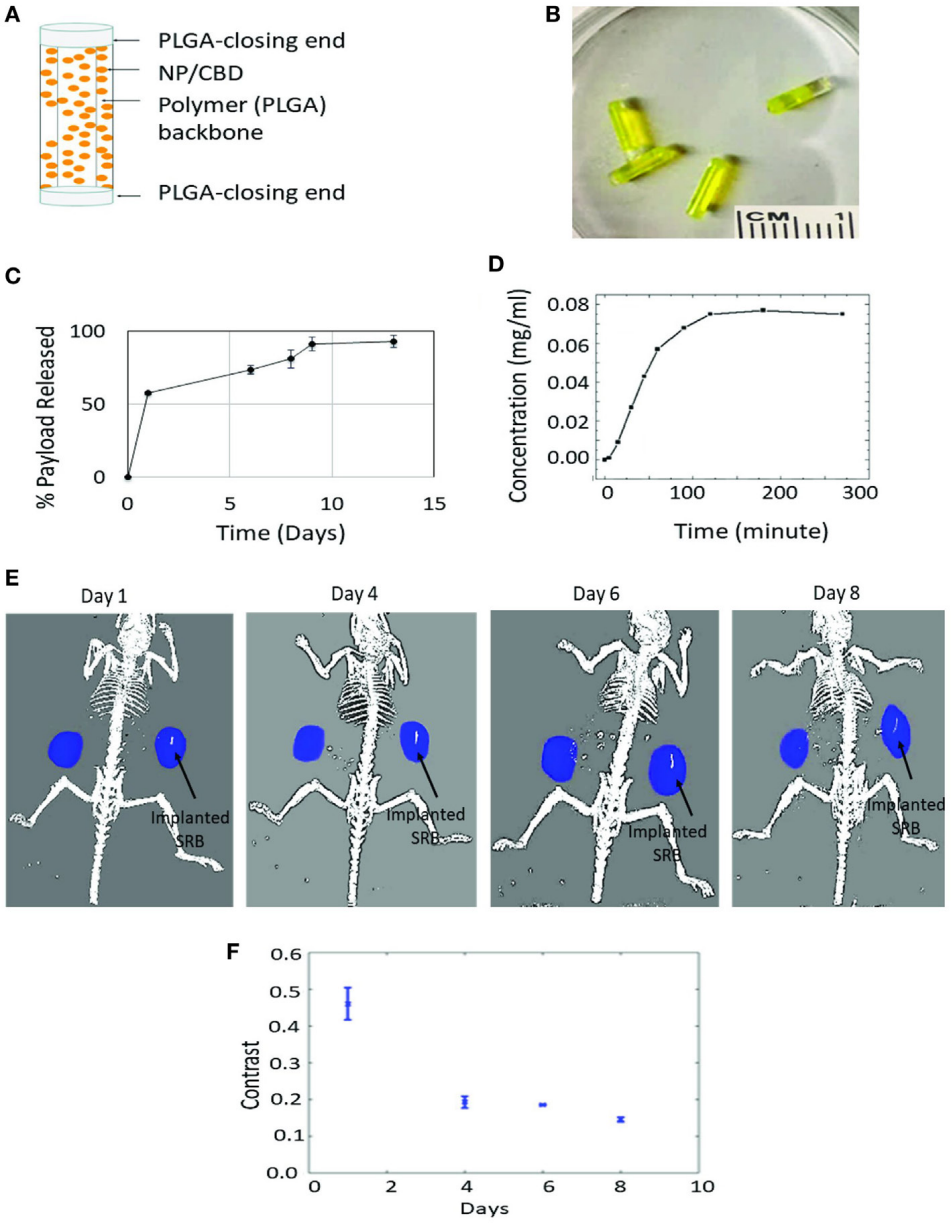
Sustained release of cannabinoid (CBD). **(A)** One design of multifunctional smart biomaterial (SRB) made of Food and Drug Administration-approved biocompatible biodegradable polymer loaded with a payload of CBDs/nanoparticles (NPs); **(B)** actual SRBs with payload; **(C,D)** *in vitro* release of CBD payload from SRB, demonstrating the ability for **(C)** sustained release over many days, and **(D)** quick release. The release kinetics (i.e., how slow or fast) can be optimized to treatment schedules, by varying the degree of cross-linking in the polymer or the polymer weighting. **(E)** Computed tomography image of mouse imaged over time with small animal radiation research platform showing degradation of SRB as payload is released over time in the tumor on the right side of image. **(F)** Average pixel intensity from maximum amplitude image for all image slices. The data are obtained from evaluating the same region of interest in the processed maximum intensity images.

The design of the smart biomaterial is illustrated in Figure 3A, while actual SRBs with CBD payloads are shown in Figure 3B. The result in Figure 3C shows the sustained release of the payload over time. Noteworthy is the initial burst release with over 50% of the payload released over the first day, followed by a slower prolonged release over several days. Such sustained release is designed to expose tumor cells to the CBDs over a prolonged period to enhance therapy outcomes.

Figure 3D shows quicker release results demonstrating potential for controlled release, e.g., by varying the weighting of the SRB polymer component or degree of cross-linking of the smart polymer, to allow for quick or slow release as needed for any treatment schedule. Such controlled/customizable release may be useful when combining CBDs with other treatment modalities like RT, chemotherapy, or immunotherapy. Meanwhile, Figure 3E shows processed CT images of mice with tumors on both flanks, with one of the tumors on the right side (image view) implanted with the SRB. The decrease in intensity of this maximum amplitude image indicates that overtime the SRB degrades to release the payload into the tumor subvolume (Figure 3F).

**Figure 4:**
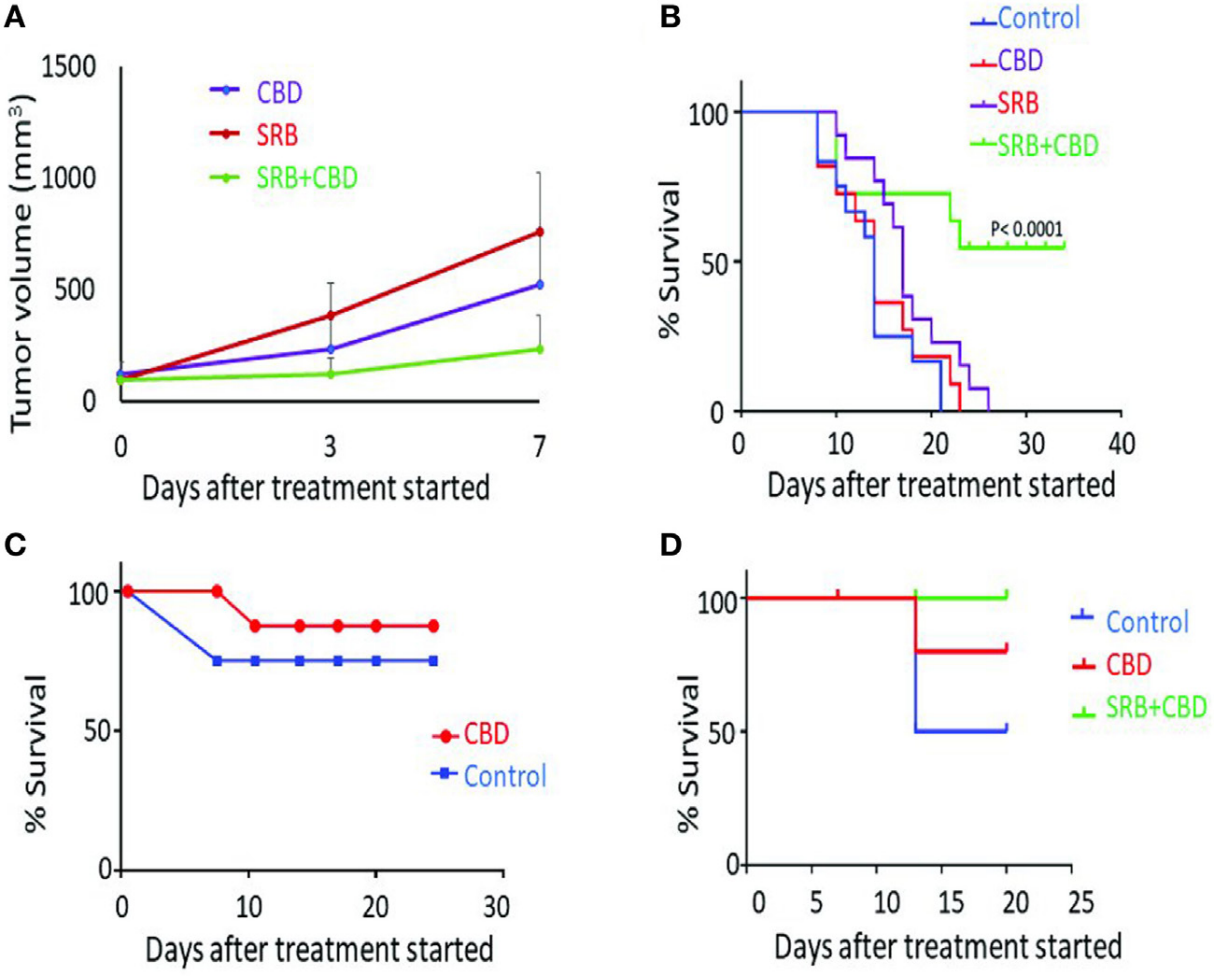
Antitumor effect of cannabinoid (CBD) and smart biomaterial (SRB)-CBD *in vivo*. **(A)** Lung tumor volume measurements highlight greater inhibition of tumor growth when using CBD-loaded SRBs compared to direct intratumoral administration (IT injection) and control cohorts: empty SRB. **(B)** Inhibition of lung tumor growth when using SRBs for sustained delivery of CBD, confirming higher killing effect of sustain release of CBD in the tumors. The Kaplan–Meier survival curve showing survival time (in days) for mice tumors treated with an empty SRB (*n* = 11), direct CBD (*n* = 13), CBD-loaded SRB (*n* = 12), and control cohort (*n* = 12). **(C,D)** A pilot study showing increased survival benefit of pancreatic tumor treated with **(C)** direct IT injection of CBD compared to control and **(D)** using SRBs for sustained delivery of CBD.

Further confirmation of the release of CBD is evident in its action in Figure 4A, which shows tumor growth inhibition results in animal cohorts with CBD-loaded SRB compared to cohorts with direct IT administration of the same dose of CBD, versus control cohorts. Results of separate survival study also supports this (Figure 4B) comparing mice treated with empty SRBs (*n* = 11), mice treated with CBD (*n* = 13), versus mice treated with CBD-loaded SRB (*n* = 12). This result shows significantly (*p* < 0.0001) increased survival for the mice with CBD-loaded SRBs relative to the other cohorts including control cohort (*n* = 12). These initial lung tumor survival study results support the hypothesis that sustained delivery with prolonged exposure of tumor cells to CBD may be more effective in inhibiting tumor growth than direct IT administration of the same dose of CBD.

Figures 4C,D show initial results from a parallel study with pancreatic tumors in mice. Direct IT injection of CBD demonstrates slight increase survival benefit compared to untreated mice (Figure 4C). In another study, the benefit of prolonged exposure of tumors to CBD is again observed in Figure 4D with 100% of the mice alive in the CBD-SRB cohort, compared to fewer mice alive in the CBD (IT) and control group. The result here justifies further studies with higher concentrations of CBDs delivered by the SRBs which could further enhance tumor cell kill.

## Discussion

Pancreatic cancer is one of the deadliest cancers, with a dismal 5-year survival rate of less than 5% (24, 25). Meanwhile lung cancer is amongst the top killers, with a growing burden, especially in low- and middle-income countries with limited access to treatment (19). There is, thus, great need to develop more effective and accessible therapeutic approaches for treating these cancers. Our results suggest that the use of a combination of strategies could allow for greater therapeutic efficacy when using CBDs for cancer treatment. The *in vitro* study results showing synergistic outcomes when using CBDs in combination with RT are in consonance with previous work highlighted in recent reviews (1, 2). In one such previous study, synergy was observed *in vivo* when using RT with CBDs in the treatment of brain tumors, in a glioma model (26). Over the years, different reports have advocated for investigations of combination therapy approaches where such synergies could be established toward clinical translation (1, 13).

A significant innovation in the approach to leverage CBDs for cancer treatment highlighted by our study is the use of smart biomaterials loaded with CBDs. In general, smart materials (14) are designed to be sensitive to specific stimuli (e.g., tumor microenvironment, pH, the wavelength, or intensity of incident radiation or an electrical or magnetic field). Once activated, they can respond in active ways including changing their structure to deliver payloads (e.g., CBDs in this case), or other functions that have the potential to effectively enhance treatment outcomes. Advancing a smart CBD cancer therapy approach with smart biomaterials presents a number of advantages toward enhancing therapeutic efficacy. First, the sustained delivery of CBDs *via* this approach will allow for prolonged exposure of the tumor cells to CBDs with expected enhanced effectiveness in tumor cell kills as seen in our initial results. Highlighting the viability of such a sustained delivery approach, the US Food and Drug Administration (FDA) has approved biodegradable disks infused with carmustine for the treatment of brain tumors (27) for greater effective treatment outcomes. Our approach with SRBs could also be developed to leverage the antitumor effects of CBDs more effectively.

Second, the *in situ* delivery afforded using smart biomaterials allows direct delivery of CBD payloads to the tumor subvolume while minimizing off-target toxicities, as seen with other delivery approaches like oral or intravenous administration. The *in situ* delivery also ensures that 100% of the payload is delivered to the tumor compared to other approaches. This could allow for reducing the dose of CBDs used, to further minimize any potential toxicities or side effects which have so far limited clinical translation. CBD receptors are not located in the brainstem areas which control respiration and, therefore, lethal overdoses from CBDs are not common (8, 13). Nevertheless, there are CBD receptors in other tissues throughout the body, which may lead to toxicities or adverse effects such as tachycardia, hypotension, conjunctival injection, bronchodilation, and decreased gastrointestinal motility. Site-targeted delivery *via* SRBs could minimize such adverse effects as well as the psychoactive effects that have limited clinical translation efforts. Ongoing work is focusing on demonstrating this expected toxicity advantage in a large cohort study.

A limitation of the current study is the short-term investigation on the survival. This is partly due to an initial focus to explore and demonstrate feasibility to inform further studies. With view to clinical translation, further investigations will build on the current work for longer term survival studies employing CBD-loaded smart biomaterials with and without RT. This will also include investigations of other CBDs besides CBD that have demonstrated potential as anticancer agents but have not been rigorously tested, or have been limited by off-target toxicities, which may be minimized with the use of SRBs.

**Figure 5:**
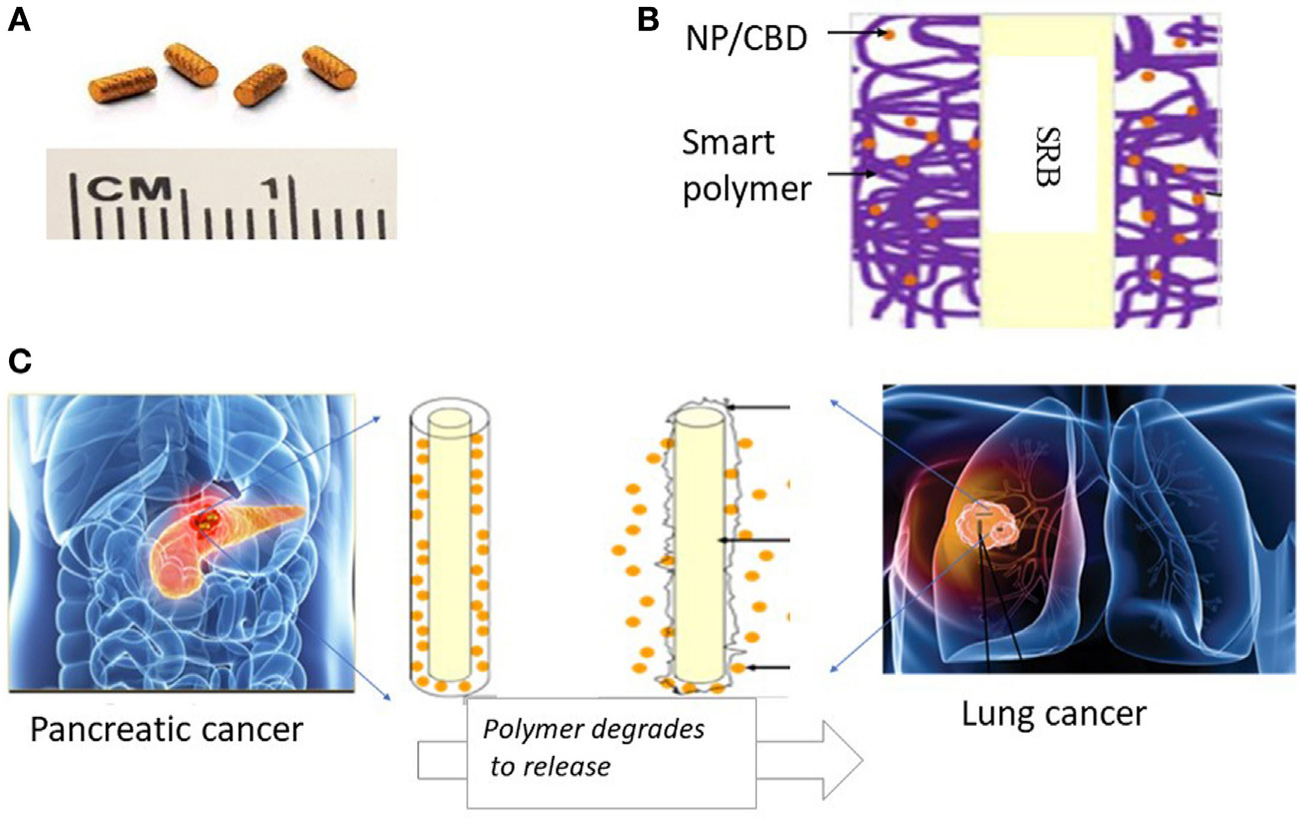
Illustration of innovative approach with potential to significantly enhance therapeutic efficacy using cannabinoids (CBDs). **(A)** Currently used commercially available inert radiotherapy (RT) biomaterials, e.g., fiducials (CIVCO Medical); **(B)** one design of multifunctional biomaterial [smart biomaterial (SRB)] made of Food and Drug Administration-approved biocompatible biodegradable polymer loaded with a payload of CBDs; **(C)** potential clinical translation pathway is envisioned where the smart SRB could simply replace the inert biomaterials currently used for image-guided RT. Such replacement would come at no additional inconvenience to cancer patients. Once in place the SRB can be activated to sustainably release its payload as the polymer coating degrades for greater effective tumor cell kill working in synergy with RT as highlighted by our study results.

Figure 5 illustrates a potential pathway for clinical translation using SRBs that integrates and builds on the results of this study, demonstrating the benefits of combining CBDs with RT and smart biomaterials. In current clinical practice, inert RT biomaterials (fiducials, spacers, beacons) illustrated in Figure 5A are routinely employed to ensure geometric accuracy during RT for tumors that move during treatment, e.g., lung or pancreatic tumors due to respiratory motion (28). We propose that these inert RT biomaterials could simply be replaced by CBD CBD-loaded SRBs (Figure 5B), at virtually no additional inconvenience to cancer patients. The multifunctional SRBs will be able to ensure geometric accuracy but also sustainably deliver potent CBD payloads directly to the tumor with the anticipated benefits of greater therapeutic efficacy and minimal toxicities. While our initial studies have focused on pancreatic and lung tumors (Figure 5C), this approach could be extended to other sites where inert RT biomaterials are also currently used, including: breast, prostate, and liver cancers. In the previous clinical trial on brain tumors (3), CBDs had to be repeatedly administered. Our approach could allow for sustained delivery, which will obviate the need for repeated administration and be more convenient for patients.

The use of smart RT biomaterials for sustained delivery could also integrate the use of NP drones (12) loaded with CBDs that can bind specifically to tumor cells to deliver their potent payloads, enhancing tumor cell kill with minimal off-target distribution. The drone technology could also be designed to target CBDs to CB1-type receptors expressed on the peripheral terminals of nociceptors around the RT planning target volume for CBD-induced analgesia. It has been shown (29) that CBDs mediate analgesia largely *via* peripheral type 1 CBD receptors in nociceptors, so such an approach with sustained delivery could also help in pain management for cancer patients.

In the US, an increasing number of states have now legalized the use of medical cannabis. This trend has also spread internationally, with more countries recognizing the medicinal components of cannabis and legalizing medical use. Unfortunately, medical cannabis research has been lagging in animal or human placebo-controlled studies addressing barriers to clinical translation. Viable pathways to clinical translation for cancer treatment should include combination approaches or smart CBD cancer therapy that leverages the antitumor effects of CBDs with high therapeutic efficacy and minimal side effects.

Altogether, our results offer an approach for leveraging the antineoplastic activity of CBDs to achieve enhanced therapeutic efficacy during cancer treatment with the possibility of addressing toxicity concerns that have hampered clinical translation efforts. The potential for using smart RT biomaterials, which integrate enhanced tumor cell killing when combining CBDs with RT, and delivery with smart biomaterials, provide a promising pathway for clinical translation. To this end, ongoing work is investigating such SRBs loaded with CBDs, which could simply replace currently used inert RT biomaterials during image-guided RT, all at no additional inconvenience to cancer patients.

## Ethics Statement

This study was carried out in accordance with the recommendations of Dana-Farber Cancer Institute Institutional Animal Care and Use Committee (IACUC). The protocol was approved by the Dana-Farber Cancer Institute IACUC.

## Author Contributions

SY-K designed the work, acquired and analyzed data, and participated in writing the manuscript; MM acquired and analyzed data and revised the manuscript; RM and NS analyzed the CT image data and contributed in revision of the manuscript; RD and AH contributed to the concept and design of the work, reviewed and revised the manuscript; and WN contributed to the concept of the work, reviewed and revised the manuscript.

## Conflict of Interest Statement

Coauthors RD and AH work for the for-profit company Cannabis Science Inc. All other authors declare that the research was conducted in the absence of any commercial or financial relationships that could be construed as a potential conflict of interest.
